# Guy's Hospital Reports

**Published:** 1891-12

**Authors:** 


*Guy's Hospital Reports.* Edited by N. DAVIES-COLLEY, M.A., M.C., and W. HALE WHITE, M.D. Vol. XLVII., being Vol. XXXII. of the third series. Pp. xliii.—368. London: J. & A. CHURCHILL. 1890.

The present volume opens with a very readable article on the career of the late Sir William Gull, and contains several others which give evidence of useful work done chiefly by the junior members of the Guy's staff.

We would especially mention the article on "The Pathology of Acute Infective Periostitis and Acute Necrosis of Growing Bone," by A. H. Tubby, M.S.; and one by Percy C. Evans, M.B., entitled "Experiments on some Antiseptics and Disinfectants." Both of these are the result of considerable labour and experiment, and are full of suggestive thoughts.

